# Augmentative and alternative communication intervention for in-patient individuals with post-stroke aphasia: study protocol of a parallel-group, pragmatic randomized controlled trial

**DOI:** 10.1186/s13063-021-05799-0

**Published:** 2021-11-24

**Authors:** Li Huang, Szu-Han Kay Chen, Shutian Xu, Yongli Wang, Xing Jin, Ping Wan, Jikang Sun, Jiming Tao, Sicong Zhang, Guohui Zhang, Chunlei Shan

**Affiliations:** 1 Department of Rehabilitation Medicine, Yueyang Hospital of Integrated Traditional Chinese and Western Medicine, Shanghai University of Traditional Chinese Medicine, No. 110 Ganhe Road, Hongkou District, Shanghai, 200437 China; 2 Department of Communication Sciences and Disorders , University of New Hampshire, 4 Library Way, Hewitt Hall, Room 144, Durham, NH USA 03824; 3 Engineering Research Center of Traditional Chinese Medicine Intelligent Rehabilitation , Ministry of Education, 1200 Cailun Road, Pudong New District, Shanghai, 201203 China; 4 Department of Rehabilitation Science, Faculty of Education, East China Normal University, 3663 North Zhongshan Road, Putuo District, Shanghai, 200062 China; 5 School of Rehabilitation Science, Shanghai University of Traditional Chinese Medicine, 1200 Cailun Road, Pudong New District, Shanghai, 201203 China

**Keywords:** Aphasia, Stroke, Augmentative and alternative communication, Speech-language therapy, Randomized controlled trial

## Abstract

**Background:**

People with post-stroke aphasia commonly receive speech-language therapy (SLT) when they are admitted to hospitals. Commonly, these patients reported communication difficulties in in-patient settings. Augmentative and alternative communication (AAC) has been reported as an effective treatment approach to improve communication effectiveness, language performance, decreasing depression, and improving quality of life for this population. However, little evidence has demonstrated the use of AAC intervention (AACT) in early recovery from people with post-stroke aphasia in in-patient rehabilitation settings for improving these patients’ communication effectiveness. The pilot randomized controlled trial (RCT) will explore the effectiveness and feasibility of including AACT in regular SLT for in-patient people with post-stroke aphasia.

**Method:**

This pilot RCT is a single-blind, randomized controlled trial with two parallel groups. Both groups receive a 1-h treatment session, including either both AACT and SLT or SLT only for ten consecutive days. We aim to include 22 in-patient participants with post-stroke aphasia in each group. Participants will be assessed at pre- and post-intervention and 2 weeks after intervention. The primary outcomes are the ability of communication measured by the communication of basic needs subtest in the Functional Assessment of Communication Skills for Adult (FACS) and the overall language performance measured by the Chinese Standard Aphasia Battery (ABC). The secondary outcomes include a 10-min conversation, the 10-item Hospital version of the Stroke Aphasic Depression Questionnaire (SADQH-10), the Stroke-Specific Quality of Life Scale (SS-QOL), and a patient and caregiver satisfaction questionnaire.

**Discussion:**

This pilot RCT will contribute to new scientific evidence to the field of aphasia rehabilitation in early recovery during the in-patient period. The paper describes the trial, which will explore the effect of combining AACT and SLT and SLT only, our choice of primary and secondary outcome measures, and proposed analyses. The study results will provide information for implementing AACT in the regular in-patient SLT of future RCTs.

**Trial registration:**

Chinese Clinical Trial Registry database (ChiCTR) ChiCTR2000028870. Registered on 5 January 2020

**Supplementary Information:**

The online version contains supplementary material available at 10.1186/s13063-021-05799-0.

## Background

Stroke is a leading cause of disability worldwide [1]. Nearly one-third of patients with stroke have post-stroke aphasia [2–4], which is defined as an impairment of the complex process of interpreting and formulating language symbols affecting auditory comprehension, reading, and oral-expressive language and writing [2, 4]. People with aphasia commonly experience different levels of expressive language impairments, ranging from occasional word-finding difficulties to severe verbal communication difficulties [4]. These language and communication difficulties often affect patients’ emotions and quality of life [5–8].

When people with aphasia are admitted to hospitals after stroke, they usually receive speech-language therapy (SLT) to improve their language and communication abilities [4, 9, 10]. However, they often reported difficulties in expressing their daily-life and medical needs because of aphasia. Meanwhile, caregivers and healthcare professionals also reported difficulties when communicating with people with aphasia [11]. Therefore, besides SLT, augmentative and alternative communication (AAC) becomes a solution for the issue. AAC is a rehabilitation approach that helps people with aphasia communicate with others [12–14]. People with aphasia can use aided AAC systems (e.g., communication books or high-tech computer devices) to communicate basic needs, deliver information, maintain social closeness, and use social etiquette [12, 15].

Historically, AAC intervention (AACT) was primarily used to support people with aphasia’s communication needs as a compensatory strategy but was not used to restore their language abilities [14–18]. Currently, few studies reported that AACT could facilitate language recovery. Several AAC treatment studies have documented people with aphasia’s improvements in overall language performance and spoken discourses to unfamiliar listeners [19–21]. Moreover, people with aphasia also showed the ability to use the letters on an AAC device or a communication board to facilitate spoken language during communication breakdowns [20, 22]. However, there is still a lack of evidence of the effectiveness of AAC practices in in-patient rehabilitation settings. Thus, an understanding of AAC practices and supports related to serving individuals with post-stroke aphasia in in-patient rehabilitation settings is essential to develop evidentiary AAC intervention in the specific setting.

It is also important to note that there is limited evidence of AACT for Chinese-speaking people with aphasia. People with aphasia in in-patient rehabilitation settings in China are mainly in acute rehabilitation or subacute facilities. Their communication demands include expressing basic and medical needs (e.g., asking a nurse to call a family member). They need to share information and feelings with their family members and interact with medical professionals about needs and concerns with regard to their healthcare. Evidence indicated that this population could learn to use yes/no cards and communication boards with pictures and words for different communication needs [12]. While the previous studies supported the concept of using AAC in hospital settings [8, 11], there is still no evidence to support this approach in Chinese-speaking people with aphasia. Therefore, an AACT RCT study is needed to investigate the feasibility of the AACT and build up the foundation of research evidence in Chinese communities.

In addition, previous studies have reported that people with aphasia and their families often reject to adopt AAC strategies with the fear that AAC may interfere with or impede the restoration of their natural language system [14, 19, 23]. Similar concerns are commonly raised in the clinical settings in China. However, studies have shown that AACT may simultaneously strengthen communication by reducing the pressure to retrieve target concepts using impaired language functions [20]. Since the present study aims to introduce a new approach (AACT) in an in-patient rehabilitation setting, considering the potential concern of people with aphasia and their families becomes essential. Therefore, instead of comparing AACT and SLT exclusively, the present study aims to compare AACT’s effect along with regular SLT to SLT only in individuals with post-stroke moderate to severe aphasia in an in-patient rehabilitation program.

### Objective

The present study focuses on the effectiveness of AACT combined with SLT for individuals with moderate to severe aphasia in in-patient rehabilitation settings. The primary aim is to evaluate whether people with aphasia who receive AACT with SLT have more functional communication and language performance improvement than those who received only SLT. The secondary aim is to evaluate the improvements in depression symptoms and quality of life after receiving AACT and SLT only.

## Methods

### Study design and procedure

The study is a single-blind, pragmatic, pilot randomized controlled trial with two parallel groups. According to the PRECIS-2’s domains, the study is considered rather pragmatic because of the following characteristics [24]. People with moderate to severe aphasia usually receive speech-language therapy as part of usual care in in-patient settings in China. The participants of the study will be recruited from the study site from in-patient patients. When patients with aphasia are admitted in hospitals, they usually receive speech-language therapy daily for at least 2 weeks. The study design is based on the usual SLT schedule and extended the duration per treatment session for adding the AAC intervention in usual care. The primary outcome and analysis of the study is the improvement of functional communication which is also the essential outcome in the usual care.

Participants will participate in a 1-h treatment session/day for ten consecutive days. During each session, participants will receive 30 min of AACT in addition to 30 min of SLT (experimental group) or 60 min of SLT (control group). Participants in the AACT + SLT group will learn to use a paper-made communication board to express their needs. The communication board includes 45 pictures relating to basic needs, emotional expression, medical conditions, and daily activities. Participants’ regular rehabilitation interventions, such as physical and occupational therapies, remain the same during the experimental period.

**Fig. 1:**
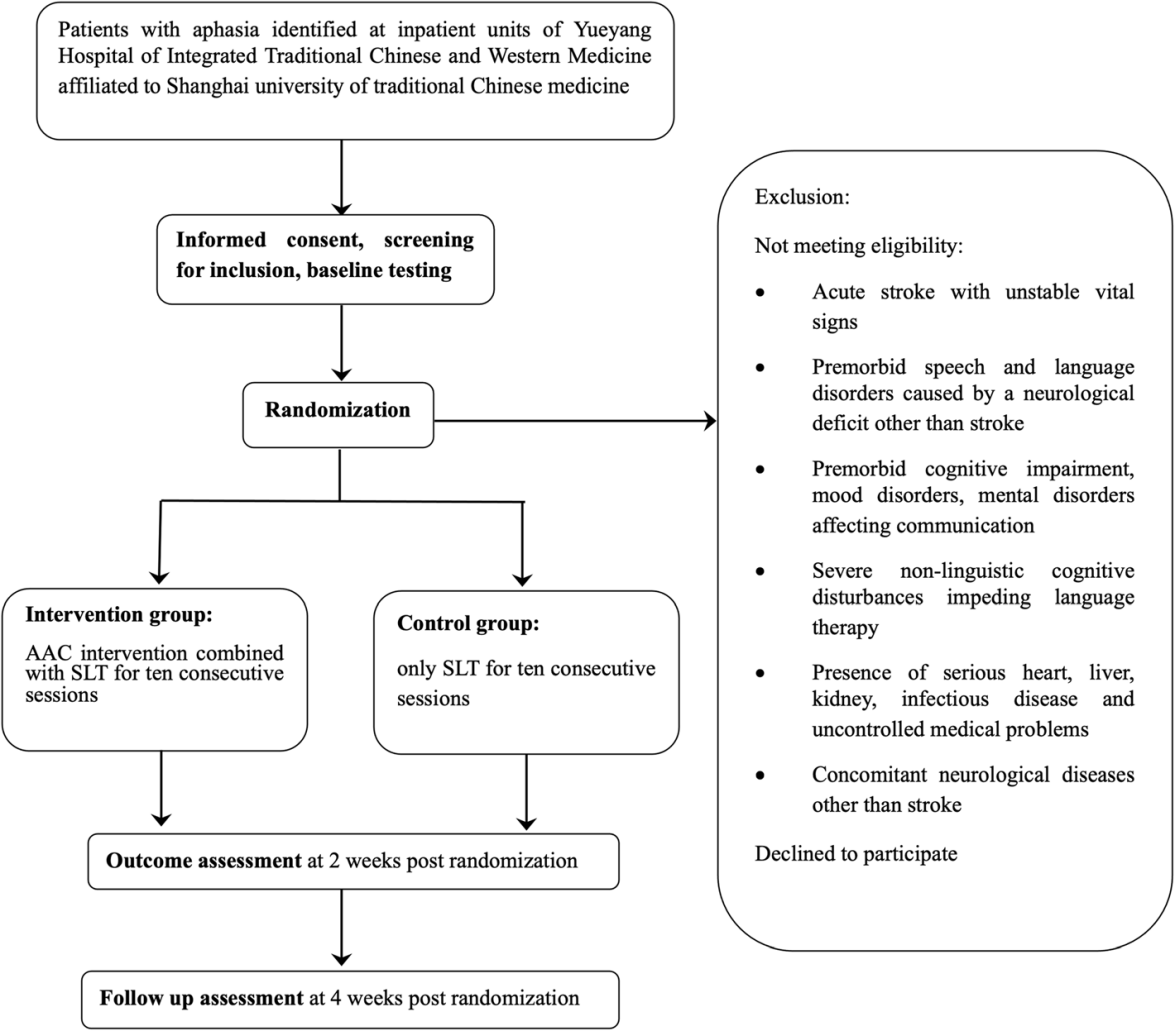
Flow diagram trial design

The participants will be evaluated at pre-intervention (prior to randomization) for collecting the demographic and baseline data. The post-intervention evaluation will be completed after participants complete the 10-day training, which is 2 weeks after randomization. Last, a 2-week follow-up will be conducted after the post-intervention evaluation (see Fig. 1).

### Ethics approval

This study has been approved by the Medical Ethics Committee (MEC) of YY hospital (reference number: 2019-118, approval received in November 2019). The researchers will report serious adverse events to the MEC. The events will be managed based on institutional policies.

Any modifications to the protocol which may impact on the conduct of the study, potential benefit of the patient, including changes of study objectives, study design, patient population, sample sizes, study procedures, or significant administrative aspects, will require a formal amendment to the protocol. The PI will first notify the IRB board of the YY hospital if there is any modification needed. Such amendment will be agreed upon by the research administration center of YY hospital and approved by the Ethics Committee of YY hospital prior to implementation and notified to Shanghai University of Traditional Chinese Medicine in accordance with local regulations. Revised protocol will also be updated in the Chinese Clinical Trial Registry database (ChiCTR) as ChiCTR2000028870.

### Participants

People with aphasia who are receiving in-patient stroke rehabilitation services in Yueyang Hospital of Integrated Traditional Chinese and Western Medicine Shanghai University of Traditional Chinese Medicine, Shanghai, China (YY hospital), are screened for eligibility. The physician in the in-patient department who is in charge of the patients’ rehabilitation service will recruit the patient and provide written information and oral explanation to the participants and their family members. The SLP who is in charge of evaluation assists the physician for diagnosing aphasia and the severity of oral production. Participants are enrolled by the physician according to the inclusion and exclusion criteria as listed in Table 1. All participants and their family members are required to review the information and sign the consent form before participating in the study.

**Table 1:** Inclusion and exclusion criteria

**Inclusion criteria** 1. Diagnosis of aphasia, after stroke, as confirmed by a qualified speech-language therapist ascertained with the Chinese Standard Aphasia Battery (ABC) test [25] 2. Moderate to severe information reduction of verbal production (percentile score of 50 or lower on the ABC verbal production subtest) 3. First onset of stroke, anytime post-stroke 4. Age 20–80 years 5. Mandarin Chinese as the first language 6. Able to participate in intensive therapy **Exclusion criteria** 1. Acute stroke with unstable vital signs 2. Premorbid speech and language disorders caused by a neurological deficit other than stroke 3. Premorbid cognitive impairment, mood disorders, and mental disorders affecting communication 4. Severe non-linguistic cognitive disturbances impeding language therapy 5. Presence of serious heart, liver, kidney, infectious disease, and uncontrolled medical problems 6. Concomitant neurological diseases other than stroke	

Once a participant is enrolled or randomized, the study site will make every reasonable effort to follow the participant for the entire study period. The research team are responsible for implementing standard operating procedures to maintain interest in the study and achieve follow-up, including regular reminders for participants and their caregivers. Since the study is conducted in an in-patient setting, the research team anticipate the retention will be good except that participants have urgent medical conditions occurring during the treatment period. Participants may withdraw from the study for any reason at any time. The investigator also may withdraw participants from the study if they are unwilling to comply with required study procedures after consultation with the Protocol Chair in YY hospital.

### Randomization and blinding

In-patient participants in YY hospital who meet the inclusion criteria will be randomly assigned to the experimental or control groups after the pre-intervention assessment. A researcher who is not involved in the study will generate a randomization sequence list based on the ratio of 1 to 1 for two groups. Group allocation will be obtained at the beginning of the first intervention session. The SLP in charge of all intervention sessions will open an opaque envelope that includes the assigned allocation. Another SLP who does not know the treatment allocation will conduct all the participants’ pre- and post-intervention assessments, and pre-intervention assessment will be performed before group allocation. The SLP performing the intervention is not blinded to group allocation because of the nature of the intervention, while the participants are blinded to allocation since they only know that the intervention is to help them improve communication, and can hardly distinguish AAC from SLT. Data analysis will be conducted by a data analyst who is blinded to group allocation.

### Intervention

#### Control group (SLT only)

The participants in the SLT only group will receive a 1-h regular SLT per day for ten sessions. Fifty words related to the stroke rehabilitation program’s routine are selected. The fifty words include categories as vegetables and fruits, daily supplies, daily actions, body parts, and the salutation of surrounding medical professionals and family members (husband/wife, son/daughter, physician, nurse, caregiver). These words are presented with a picture and a written word. The 50 words are divided into five sets (10 words/set) according to categories. Participants will practice one set every 2 days.

Participants learn the semantic and phonological knowledge of 10 words and conduct auditory comprehension (selecting target word from two/four words following verbal directions from the SLP), word repetition, and naming tasks of learned words in each session. The tasks and training procedures are the same in each session. The procedures are as follows: (1) participants are taught with the semantic and phonological knowledge of the target word one by one; (2) they are asked to repeat the word with the SLP; (3) they are asked to do auditory comprehension tasks by selecting target words from two or four cards; and (4) they are asked to name the target picture based on the Cueing Hierarchy [26].

#### Experimental group (AACT + SLT)

The experimental group will receive AACT (0.5 h) + SLT (0.5 h) per session. Participants will learn how to point to the target icons on a paper-based communication board to answer questions (e.g., “Do you have pain?”, “Where is your pain?”) in line with studies that apply AACT for people with aphasia [8, 12, 27]. The communication board contains 45 icons designed for people with aphasia to communicate with medical professionals and family members in the in-patient setting. These icons are pictures of everyday needs, clinical symptoms, types of medical care, rehabilitation services, names of people around, yes/no signs, and a rating scale, with Chinese written words on the top of each icon.

Each intervention includes three training tasks. These tasks aim to help people with aphasia learn how to use the communication board [21]. The first task is symbol identification. Participants are asked to identify each icon on the communication board following verbal directions from the SLP. At first, the SLP presents the verbal model plus a demonstration: the SLP saying “point to [target icon]” while physically modeling the desired response by pointing to the target icon on the communication board. After the modeling, participants are asked to follow the exact directions. Next, the SLP presents a verbal cue only: the SLP saying “point to [target icon],” and then requires the participant to point to the target icon independently. The second task is to answer yes/no questions. The SLP asks a yes/no question relative to a particular icon (is this a picture of [target icon]) and then asks the participant to respond to the question by pointing to the yes or no icon on the communication board. The third task is to respond to questions with a specific icon. The SLP asks an open-ended question relative to a particular icon (e.g., “if you have a headache, which icon will be selected to tell the people who are surrounding you?”). Then the participant responds to the question by pointing to the target icon.

### Measurement instruments

Table 2 provides an overview of the measurement instruments used in the study. The primary outcome measures are the scale of the Communication of Basic Needs (CBN) on the Functional Assessment of Communication Skills for Adults (FACS) [28, 29]. Communication recovery is an essential outcome when people with aphasia are in hospitals. Although professionals agree the outcome is important, there is no assessment tool in the Chinese language to evaluate the outcome. Therefore, the team translated the items of the CBN domain and its scoring scale to Chinese to measure the outcome of the AACT on communication effectiveness in a hospital setting.

**Table 2:** Measurement instruments

Measurement instruments	Number of items	Score range	Measurement target
CBN subtest on the ASHA FACS	7	0–7	Functional communication skills for basic needs
ABC test	23	0–100	Language abilities, including speaking, listening, reading, and writing
10-min conversation	4	0–5	Communication efficiency in the in-patient setting
SADQH-10	10	0–3	Depression symptoms
SS-QOL	49	49–245	Stroke-specific health-related quality of life
Satisfaction questionnaire	11	1–5	Satisfaction intervention for communication improvement from both patients and their caregivers

The FACS measures functional communication skills of people with aphasia in four domains (social communication, communication of basic needs, reading/writing/number concepts, and daily planning) and in four qualitative dimensions (adequacy, appropriateness, promptness, and communication sharing). The 7-point scale measures the levels of assistance and prompting needed in each domain, and the 5-point scale measures the response in four dimensions. The inter-rater/intra-rater reliability for the CBN domain and the overall qualitative dimension are high. The concurrent validity between the Western Aphasia Battery and the Functional Independence Measure (FIM) is moderate [28].

An informal test of a 10-min conversation is used to measure participants’ functional communication efficiency in the in-patient setting pre- and post-intervention. Conversation analysis is a common procedure in regular aphasia assessment [30]. The 10-min conversation is evaluated using the 5-point scale of the four qualitative dimensions (adequacy, appropriateness, promptness, and communication sharing) in the FACS. The results of the 5-point scale allow the team to evaluate changes of the quality of conversation pre- and post-intervention. The participants are asked to talk about their daily lives and their medical services with the SLP in the conversation. The conversation will be video recorded and analyzed by the SLP conducting the assessments.

The secondary measures include the Chinese Standard Aphasia Battery (ABC), a standard test to diagnose aphasia and determine the type of aphasia for the Chinese-speaking population and widely used in clinical practice and research in local sites, to measure participants’ overall language performance [25]. The Chinese version of the 10-item Hospital version of the Stroke Aphasic Depression Questionnaire (SADQH-10) with adequate content validity and the test-retest reliability (> 0.80) is used for evaluating depressive symptoms in people with aphasia [31, 32]. The Chinese version of the Stroke-Specific Quality of Life Scale (SS-QOL) with high stability and validity is used for evaluating the stroke-specific health-related quality of life [33, 34].

For qualitative evaluations, the first author designed a 5-point scale questionnaire to evaluate the satisfaction of the interventions for this trial. The questionnaire includes four items for both control and experimental groups, three items for both groups’ caregivers, and two extra items for the experimental group and the caregivers. The questions were reviewed by the research team to ensure the readability and appropriates. The participants and their caregivers are asked to choose from 1 (very dissatisfied) to 5 (very satisfied) to evaluate the satisfaction for materials (common cards and AAC paper board) and communication behavior with caregivers and medical professionals after the intervention.

**Fig. 2:**
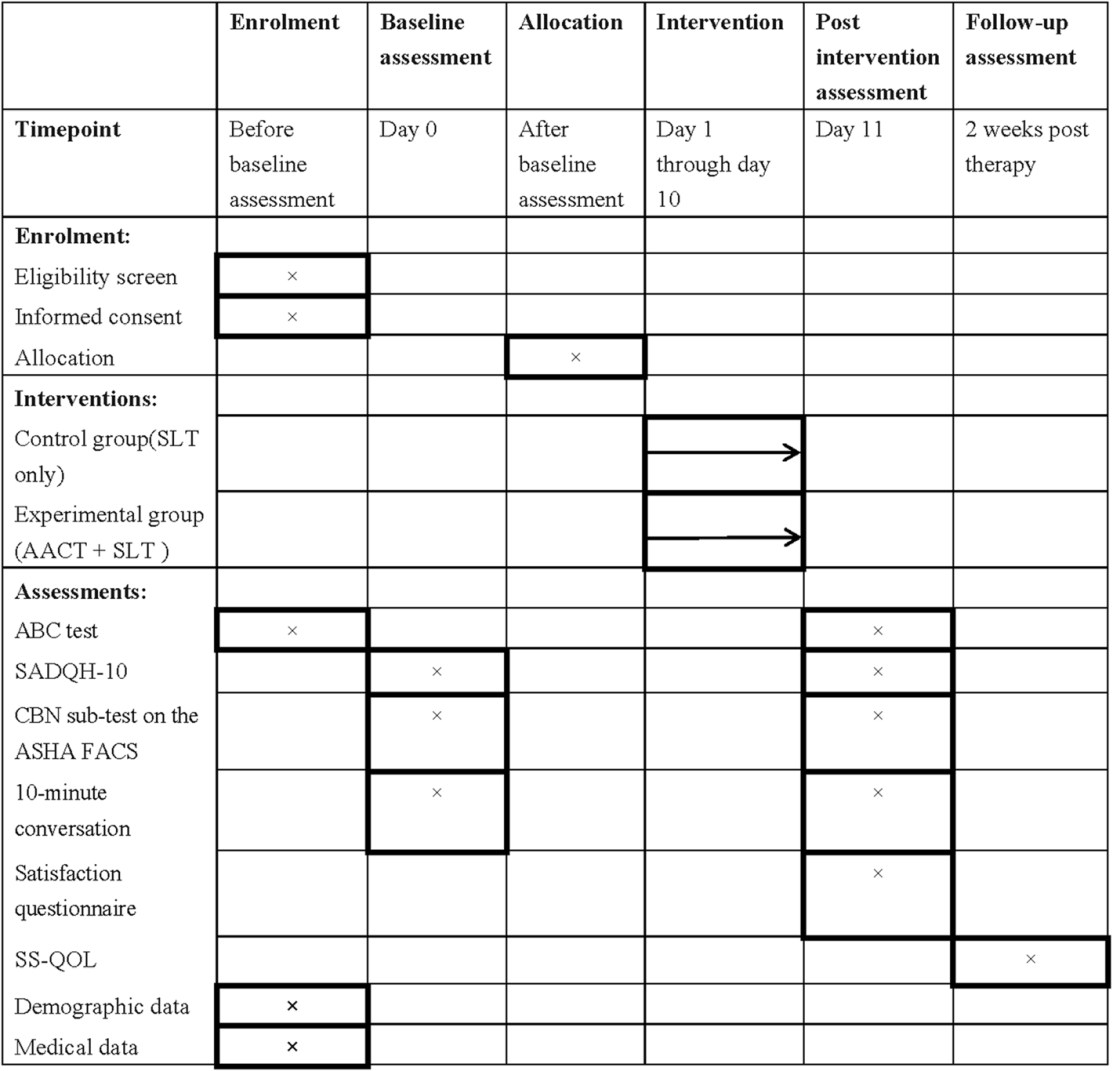
Trial schedule of enrollment intervention and assessment

Figure 2 shows the schedule of each outcome measure. The CBN on the ASHA FACS, the ABC, a 10-min conversation, and SADQH-10 are completed pre- and post-intervention. The satisfaction questionnaire is completed after the intervention. The SS-QOL is completed after a 2-week follow-up for evaluating the quality of life after participants are discharged by the in-patient rehabilitation program. Additional file 1 is an overview of the Standard Protocol Items: Recommendations for Interventional Trials (SPIRIT) checklist.

### Sample size

No large RCTs have been conducted in the field of aphasia AACT in in-patient rehabilitation settings. Previous AAC studies used interventions not applicable to the current protocol. Therefore, we are unable to calculate the prior accurate sample size. We will use the CBN score in the FACS to investigate the possible effect. We consider a minimum improvement of 1 point on the 7-point scale in FACS is clinically significant. Given an expected standard deviation of 1 based on prior norm references [28, 35], the trial must include a total of 44 participants. Each group needs 22 participants to detect this difference with a significance level of 5 and a power of 90. After considering a dropout rate of 20%, the study aims to recruit 56 participants in total. We anticipate that this pilot study will provide information for more accurate sample size calculation in the future.

### Data analysis

Outcomes of interest will be analyzed on an intention-to-treat basis. Characteristics of the sample and the descriptive statistics will be reported. The primary analysis will be conducted without adjustment. The mixed model analysis will be used to analyze the continuous repeated-measure data because of its advantages of accommodating missing data and the flexibility in small samples and treating time. Data will be examined for differences between groups and over time. Post hoc tests will be performed to explore factors including age, sex, education, and initial stroke/aphasia severity, considered to affect the trial’s outcome. The data from conversation samples and the satisfaction survey will be suitably coded for qualitative responses to be categorized and examined.

## Discussion

This protocol describes a pragmatic pilot randomized controlled trial that can provide a valuable contribution to people with post-stroke moderate to severe aphasia in in-patient rehabilitation settings. The trial’s primary objective is to investigate the effectiveness of AACT combined with traditional SLT in communication/language recovery. To our knowledge, no RCTs have explored the combination of two treatment approaches for people with aphasia in in-patient rehabilitation settings.

This RCT will look at the feasibility of scaling the AACT up for larger trials and clinical implementation. Therefore, we aim to have a larger sample size compared to previous studies. While AACT has been developed several decades in developed countries, it is still relatively new for most developing countries, including China. Future studies may apply the results of the study to them if the trial is feasible. People with post-stroke aphasia commonly receive traditional SLT that aims to recover their verbal communication skills through behavior modification, cognitive therapy, and pragmatic therapies. However, many of them face immediate communication challenges when they are admitted to hospitals. Evidence suggests that people with aphasia have decreased quality of life, poorer health outcomes, and limited participation in medical encounters due to their communication and language disabilities [8]. These people are more likely to experience adverse medical events (e.g., medication errors) than those without a communication disability [8, 36]. AACT can be the solution to improve the situation. People with post-stroke aphasia can use AAC to support their communication effectiveness when spoken communication skills do not work [21].

Our trial trains people with aphasia to use a paper-based communication board with caregivers and healthcare providers in in-patient rehabilitation settings. It is easy to access and cost-effective, which is essential for all clinical settings. As an in-patient stay is often the first step on a long-term care continuum, AAC can maximize people’s communication skills with aphasia within desired life roles and prepare them and their families for life after the in-patient stay [8]. When the trial is completed, the results will also give us a better understanding of the AACT’s dual role in simultaneously driving inter-systemic reorganization with the potential to support language function while providing compensatory strategies during communication breakdowns in in-patient settings.

In our previous experience, AAC is often not prescribed in the early stages of recovery because patients with aphasia and their family members are worried that receiving AACT will impede language recovery [19]. It is a common AAC myth in clinical settings. However, recent evidence suggests that early AACT in in-patient settings may increase AAC acceptance and decrease the risk of not using language during the early recovery stage. Early AACT may also increase people with aphasia’s motivation to communicate in various situations and have better long-term participation outcomes. Therefore, they were more active when making their healthcare decisions, experiencing increased participation in life events, and discovering new social roles [19]. This evidence supports the purpose of the trial’s intention to investigate early AACT’s effectiveness in in-patient settings. The results of the trial will also contribute to the research evidence of AACT in Chinese-speaking people with aphasia.

A limitation of this study is that this protocol is not double-blind. However, the participants and the SLP will be blinded to treatment allocation. The study also cannot eliminate the confounding effects from the early-stage spontaneous recovery after stroke. The trial is designed to investigate the effectiveness of the current practice protocol in the local site. Therefore, the follow-up is short. Since the research evidence of the effectiveness of AACT in in-patient settings is limited, the trial’s results will also provide new insight into implementing AACT in actual clinical practice.

## Trial status

This trial is registered in the Chinese Clinical Trial Registry database (ChiCTR) as ChiCTR2000028870 on 5 January 2020, and the current protocol is the first version. The recruitment of the trial started on 20 January 2020, and delay the completion time until December 2022 as reported in the ChiCTR database due to the research site’s restriction of admitting patients because of COVID-19. Fourteen participants have been recruited and randomized into the trial until now.

## Supplementary Information

**Additional file 1.** Overview of the Standard Protocol Items: Recommendations for Interventional Trials (SPIRIT) checklist

## Data Availability

The datasets analyzed during the current study are available from the corresponding author on reasonable request.

## Abbreviations

AAC: Augmentative and alternative communication
ABC: Aphasia Battery of Chinese
ASHA: American Speech and Hearing Association
CBN: Communication of Basic Needs
CONSORT: Consolidated Standards of Reporting Trials
FACS: Functional Assessment of Communication Skills for Adult
MEC: Medical Ethics Committee
RCT: Randomized controlled trial
SADQH-10: 10-item Hospital version of the Stroke Aphasic Depression Questionnaire
SLP: Speech and language pathologist
SLT: Speech-language therapy
SPIRIT: Standard Protocol Items: Recommendations for Interventional Trials
SS-QOL: Stroke-Specific Quality of Life Scale
YY hospital: Yueyang Hospital of Integrated Traditional Chinese and Western Medicine Affiliated to Shanghai University of Traditional Chinese Medicine
